# Factors Associated with Health-Related Quality of Life among Older Adults in Rural South Korea Based on Ecological Model

**DOI:** 10.3390/ijerph19127021

**Published:** 2022-06-08

**Authors:** Shinae Lee, So Hyoung Hong, Hye Young Song

**Affiliations:** 1 Department of Nursing, Daegu Health College, Daegu 41453, Korea; shinaelee@dhc.ac.kr; 2 Department of Nursing, Gunjang University, Gunsan 54045, Korea; 3 Department of Nursing, Woosuk University, Wanju 55338, Korea; lemonbam84@woosuk.ac.kr

**Keywords:** older adults, health related quality of life, rural population, ecological model

## Abstract

As the portion of older adults in the population in rural areas of South Korea exceeds 20%, the importance of health-related quality of life is increasing. The aim of the study was to examine the health-related quality of life through the ecological model and its basic determining factors for older adults. The study was conducted on 184 respondents aged 65 and over living in rural areas of South Korea. The measurements were health-related quality of life, health care service needs, sleep quality, social support, and personal characteristics. The collected data were tested using descriptive, t-test, ANOVA, and hierarchical multiple regression. The results showed that older adults in rural areas experienced a low quality of life. Religion, having a helper, and social support were significantly related to health-related quality of life in older adults. This directly shows that the government should make efforts to build a social support system to improve the gap between urban and rural areas. To improve the health-related quality of life of older adults in rural areas, it would be helpful to increase physical activity and to form a community, leading to a social network.

## 1. Introduction

In South Korea, the older adult population aged 65 and over has been rapidly rising, from 13.1% of the total population in 2015 to 16.5% in 2021 [1,2]. The proportion of the aged population in rural areas of South Korea is now over 20%, indicating its entry into a super-aged area. It is expected to become a super-aged society by 2025 [2]. About 42% of the older adults in South Korea receive a national pension of 7.1 million won per year; however, these numbers are far lower compared to developed countries [3]. Although the basic pension scheme, including the National Pension System, was introduced in 1998, the percentage of older adults belonging to the relative poverty group is 40.4%. In South Korea, relative poverty is defined as households that receive 50% less than average household incomes. The relative risk of poverty among older adults in South Korea is the highest among major Organization for Economic Co-operation and Development (OECD) countries [4,5]. They not only face a poor economic situation, but also experience a low quality of life, including poor health [6].

Quality of life is worse in older adults who live in rural areas. Visits to hospitals are often restricted due to difficulties in transportation. If the children of older adults in rural areas have moved to the city, it is difficult for older adults to receive support and care from their families [7,8]. As a result, the quality of life is lower than in urban areas, and the problem of aging in rural areas is emerging as a serious problem [9,10]. The “Community Care Project” was introduced in South Korea, to address the needs of underprivileged older adults and to improve their health [11]. Despite such efforts to reduce the health gap between rural and urban areas, physical and mental health care systems have not been well established, resulting in a poor quality of life for older adults in rural areas [12].

In the World Health Organization (WHO), quality of life is defined as “an individual’s perception of their life within the context of the culture and values in which they live” [13]. Quality of life has been a major concern around the world, and the health-related quality of life (HRQOL) of older adults is a key goal of public health [14,15]. HRQOL is an important factor to be considered with the aging population because of its usefulness in evaluating the health and well-being of individuals and it enables the identification of health service needs [16]. HRQOL is an indicator of physical, mental, and social functioning and pain [17,18]. Sleep disturbance is a variable closely related to quality of life and is characterized by accompanying physical and psychological symptoms [19]. The continuous deterioration of sleep quality may lead to fatigue and decrease in overall quality of life, and may also cause emotional disorders, such as depression [19,20]. It is known that most older adults have poor sleep quality because of physiological changes caused by aging or disease [19]. Therefore, appropriate interventions to improve sleep quality need to be developed for the life satisfaction of old adults [20,21].

**Figure 1:**
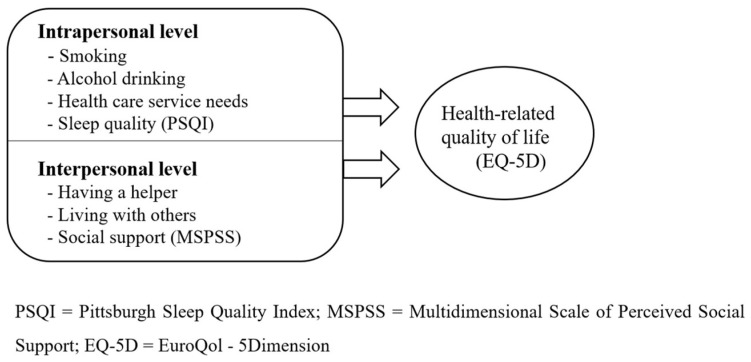
Conceptual model for predicting health-related quality of life in older adults.

Previous studies on HRQOL focused on intrapersonal factors, such as age [9], physical health [22], mental health [22,23], socioeconomic status [9], and rural life [15]. With these limited results, it is difficult to determine the HRQOL of older adults. As HRQOL is determined not only by individual characteristics, but also by relationships with family and neighbors, as well as the physical environment [24], it is important to understand the social and physical environment surrounding the individual and the context of the local community [25]. In order to understand the interaction of various factors related to an individual’s quality of life, an ecological model emphasizing the complex interaction between the individual and the environment is suitable [26,27]. This theory defines the interaction between humans and the environment as an ecological system, and has the advantage of enabling multidimensional and diverse interventions by considering the interactions between various factors affecting individual behavior [26,28]. Therefore, it is necessary to take a multifaceted approach based on an ecological model for analyzing the factors affecting the HRQOL of older adults. In particular, as old adults in rural areas have poor access to residential environments, income, and cultural services [15], it is necessary to identify not only intrapersonal characteristics but also interpersonal ones. In this study, smoking, alcohol drinking, health care service needs, and sleep quality were included as intrapersonal characteristics, while having a helper, living with others, and social support were included as interpersonal characteristics (Figure 1). 

### Aims

The purpose of this study was to identify the factors affecting the HRQOL of older adults in rural areas, based on an ecological model [20], and to provide the basic data necessary for planning a health promotion program or social support system to improve their HRQOL.

## 2. Methods

### 2.1. Study Sample and Data Collection

Participants were recruited using convenience sampling among older adults who had registered in the G-health care center located in Jeollabuk-Do, South Korea. The center is funded by the Korean government and provides health-related consulting and education services to the relative poverty group. The inclusion criteria for the study were: (1) those who are aged 65 years or older, (2) those who are able to follow instructions, (3) those who are seniors belonging to the relative poverty group in South Korea, and (4) those who voluntarily agreed to participate in the study. This study was conducted from July 1 to 15 August 2021. It took approximately 20 min to collect a paper-based survey from the participants. For those who had poor eyesight or wanted the printed questionnaire to be read out for them, the researcher directly asked the questions and noted their answers. The sample size was calculated using the G-power 3.1 program. Based on previous studies [29,30,31], when the power was set to 0.80, median effect size to 0.15, significance level to 0.05, and the number of predictors to 9 in a multiple regression analysis, the sample size was 166. Considering a 20% dropout rate, 200 people were targeted, and 184 questionnaires were included in the final data analysis, excluding 16 questionnaires that had missing answers or duplicated answers for the same question.

### 2.2. Measures

#### 2.2.1. Health-Related Quality of Life

For HRQOL, the EuroQoL 5 Dimension (EQ-5D) tool developed by the EuroQoL Group [32] was used. This tool consists of five domains: motor ability, self-management, daily activities, pain/discomfort, and anxiety/depression. Each dimension had 3 levels: no problems, some problems, or serious problems. The EQ-5D index calculates health status as a single quantitative value by assigning weights to the levels of each of the five domains [32]. The original EQ-5D was modified by customizing the quality weights considering the culture and situation of each country [33]. In this study, the modified EQ-5D which was accepted by Korea Disease Control and Prevention Agency [34], was used. The range of EQ-5D index is from −1 to 1. In previous studies [32,34] the Cronbach’s α was over 0.70. The Cronbach’s α of this study was 0.76.

#### 2.2.2. Health Care Service Needs

For measuring health care service needs, a tool developed by the Korean Ministry of Health and Welfare was used [11]. This tool comprises 9 items, with 0 points for “no” and 1 point for “yes” corresponding to the absence and presence of service needs, respectively, for each item. The highest score is 9 and a higher score indicates greater needs for health care services. This tool consists of questions about health care, nutritional status, cognitive function, mental health, abuse, loneliness and isolation, basic daily living, daily life management, and needs for care services in a residential environment. In this study, the Cronbach’s α was 0.68.

#### 2.2.3. Sleep Quality

For measuring sleep quality, a tool standardized in the Korean version [35] of the Pittsburgh Sleep Quality Index (PSQI) developed by Buysse et al. [36] was used. This tool contains 19 items, with scores ranging from 0 to 21, and higher scores indicate poorer sleep quality. A score of 5 or higher is considered as a sleep disorder. When the tool used in Buysse et al.’s study [36] was originally developed, Cronbach’s α was 0.83. In the Korean version of PSQI [35], Cronbach’s α was 0.84. In this study, the Cronbach’s α was 0.75.

#### 2.2.4. Social Support

For measuring social support, a tool retranslated by Shin and Lee [37] from the Multidimensional Scale of Perceived Social Support (MSPSS) developed by Zimet et al.’s study [38] was used. This tool consists of 12 items and each item comprises three sub-categories: family support, friend support, and special support. It is measured using a 5-point Likert scale and a higher score indicates higher social support. When this tool was developed in [38], Cronbach’s α was 0.85.

### 2.3. Ethical Considerations

We obtained prior approval for this study from the Institutional Review Boards (IRBs) of W University (No. WS-2021-25) for the ethical protection of study participants. We explained the purpose of this study and time expected for data collection, using expressions that were easy to understand. Thereafter, we obtained written consents from the participants.

### 2.4. Analysis

The collected data were analyzed using the WIN SPSS Version 25.0 program (IBM Corp., Armonk, NY, USA). The frequency and percentages were calculated to understand the general characteristics of the subjects, and the differences in HRQOL based on these characteristics were analyzed using t-tests and ANOVA. The HRQOL predictors of participants were analyzed using hierarchical multiple regression analysis.

## 3. Results

The general characteristics of the subjects in this study are shown in Table 1. The average age of the subjects in this study was 76.7 ± 7.46 years, and 71.7% of them were female. For the health-related quality-of-life (EQ-5D) level according to general characteristics, there were significant differences in education levels, religion, having a helper, and living with others. Through descriptive statistics on health care service needs, sleep quality, social support, and HRQOL, which are the main variables in this study, the levels of each variable for each participant could be identified, as shown in Table 2.

A hierarchical multiple regression analysis was performed, as shown in Table 3. In order to examine the independence test of the residuals before analysis, a Durbin–Watson’s test was performed. A result of 1.54 to 1.77 was obtained, which is close to 2, indicating no autocorrelation problem. The tolerance ranged from 0.73 to 0.97, which is larger than 0.1, and the variance inflation factor (VIF) was found in a range of 1.05 to 1.36, which is less than 10, confirming that there was no multicollinearity problem.

In Model 1, religion and education as general characteristics were input as control variables, and it was found that these two variables explained 7.5% of the dependent variable, HRQOL, with a significant impact. Religion and education were found to significantly affect health-related quality of life. In Model 2, health care needs and sleep quality as intrapersonal characteristics were added to Model 1. It was found that four variables significantly explained 14.2% of HRQOL. Religion and health care needs were found to have a significant effect on HRQOL. In Model 3, the variables of having a helper, living with others, and social support as interpersonal characteristics were added, and it was found to explain about 34.3% of the dependent variable, HRQOL. Religion, having a helper, and social support were found to be significant variables. As a result of comparing the relative influence of significant variables based on the absolute value of the standardized coefficient β, social support (β = 0.52), presence of a helper (β = 0.21), and religion (β = 0.17) were confirmed to have varying influences on HRQOL in older adults.

## 4. Discussion

This study was conducted to examine the HRQOL of older adults in vulnerable social groups and identify relevant factors affecting HRQOL. In this study, the quality-of-life score was 0.64 on average. The quality-of-life scores in existing studies conducted across Korea using the same HRQOL measurement are varied. The quality-of-life score was 0.82 ± 0.14 in Busan city [39] and 0.77 ± 0.15 in Gongju city [31], which were higher than the score in this study. The quality-of-life scores of old adults aged 60 to 74 years and those over 75 years living in China were 0.88 ± 0.15 and 0.78 ± 0.23, respectively, indicating that the quality of life of the participants of this study was very low [40]. The results of this study support the results of previous studies that living in rural areas lowers older adults’ quality of life [10,15]. The quality of life of older adults in rural areas is lower than in urban areas because of the limited access to cultural facilities and medical services [10]. Therefore, in order to improve the quality-of-life gap between urban and rural areas, it is necessary to establish a social support system to facilitate access to cultural and medical services [10].

As found in previous studies [41,42], older adults living alone showed a lower quality of life than older adults living with their family. Close friends and family members who can provide positive support influence quality of life more than demographic factors [42]. In the study [10], older adults living in homes in South India had a lower quality of life than community-dwelling older adults, and it was suggested to provide community centers for rest and recreation for older adults to improve their quality of life. Therefore, it is suggested to organize a community where older adults who live alone can maintain a close relationship with those in their surroundings.

In this study, religion was found to be a factor influencing HRQOL. Older adults following a religion had a higher HRQOL than those without a religion. This result is similar to existing research showing that religion improves quality of life by providing positive spiritual and emotional resources [43,44]. As interest in successful aging increases, especially in older adults, religion plays an important role, and the social connections through religious activities also have a positive effect on mental health [29,30].

Sleep quality has been reported as an important variable affecting quality of life, but it was not found to be significant in this study. In general, older adults over 65 years of age are 1.74-times more likely to have a shorter sleep time than those aged 19–44 [45], and as age increases, the circadian rhythm pattern of sleep shows a morning style [46]. In particular, individual differences in circadian rhythms escalate starting from the age of 65 [46], and the average age of the subjects in this study was 76.6 years old, which is on the high side. In the case of old adults, the sleep quality may deteriorate due to physiological changes associated with aging [19]. The sleep quality score of this study was 9.82, which was low. In all age groups, regular physical activity and exercise can improve not only physical health but also psychological health [47,48]. For older adults, mind–body interventions, such as Yoga, meditation, and Tai Chi, are recommended [49]. Mind–body intervention is an alternative form of exercise that consumes less energy, it is easy to match the individual’s performance level, and it is economical in terms of cost. According to the results of a meta-analysis, it was confirmed that mental and physical intervention is helpful in improving the quality of life, depression, and sleep quality of older adults [50]. Therefore, activities such as mind–body intervention can be effective for older adults with sleep disorders.

In interpersonal factors, it was confirmed that having a helper and social support significantly influence HRQOL. The HRQOL of old adults with a helper was lower than that of older adults without a helper. This can be analyzed in two ways. First, a helper is a resource for satisfying older adults’ needs, but not all relationships are positive. Spending time with someone can cause stress and conflict [48,49], and it may degrade their quality of life significantly. Second, the situation in which it is difficult to carry out daily life activities and the need for a helper itself lowers the quality of life. In the previous study, the presence of having a helper was not related to the quality of life. Instead, it was found that needing more help in performing daily living lowers quality of life [51]. As older adults need a lot of help in performing daily living activities, it is suggested that the having a helper variable be included in studies on the quality of life of older adults.

Social support was identified as the factor that had the greatest influence on HRQOL. It was consistent with the results of previous studies that the higher the social support, the higher the level of quality of life [9,31,52]. Social support is the help provided by family or friends, which promotes psychological adaptation and successful aging, thereby improving quality of life [52].

The results of this study confirmed that religion was an intrapersonal factor; having a helper and social support were interpersonal factors affecting the HRQOL of older adults in rural areas. The ecological model can comprehensively explain health behaviors influenced by individual, social, and environmental factors [28]. Therefore, when developing interventions for improving the HRQOL of older adults in vulnerable groups in rural areas, an integrated approach should be taken considering religion, having a helper, and social support as influencing factors in the study,

This study has the following limitations. First, the participants of this study were recruited from a single institution located in a rural area using convenience sampling and, thus, the representativeness of the base population could be limited. Second, there were insufficient measures of ecological models, including the morbidity of chronic diseases, diet, exercise, and activities of daily living. Therefore, in future research, it is necessary to include these variables for investigating the social networks of older adults and their access to medical facilities, corresponding to the mesosystem and exosystem, in addition to the intrapersonal factors of the ecological model for older adults living in various rural areas. Nevertheless, this study is significant in that it provides important information on the quality of life of older adults and related factors, and suggests ways to improve the HRQOL, especially for vulnerable social groups of older adults in rural areas, which is a worldwide concern.

## 5. Conclusions

This study analyzed the HRQOL of older adults in rural South Korea based on the ecological model. Older adults living in rural areas showed significantly low quality of life. Religion, having a helper, and social support were significantly related to the HRQOL of older adults in rural areas. In particular, important factors include not only intrapersonal characteristics but also interpersonal characteristics, including the environment surrounding. Thus, the government must attempt to build a social support system to mitigate the health gap between urban and rural areas. In order to improve the HRQOL of older adults in rural areas, it is important to promote and increase physical activity and form a community for the social networking of older adults.

## Figures and Tables

**Table 1:** Differences in Health-Related Quality of Life according to General Characteristics, Intrapersonal and Interpersonal Characteristics of Participants (*N* = 184).

Factors	Variables	Categories	*n* (%) or Mean ± SD	Health Related Quality of life (EQ-5D)	
				Mean ± SD	t or F (*p*)
General characteristics	Age(year)	65–69	30 (16.3)	0.70 ± 0.20	2.58 (0.078)
		70–79	83 (45.1)	0.61 ± 0.17	
		≥80	71 (38.6)	0.64 ± 0.15	
			76.66 ± 7.46		
	Gender	Male	52 (28.3)	0.66 ± 0.20	1.12 (0.265)
		Female	132 (71.7)	0.63 ± 0.16	
	Education	Uneducated ^a^	62 (33.7)	0.62 ± 0.13	4.01 (0.009) ** a < c, d; b < c
		Elementary ^b^	86 (46.7)	0.61 ± 0.16	
		Middle school ^c^	17 (9.2)	0.75 ± 0.22	
		≥High school ^d^	19 (10.3)	0.69 ± 0.23	
	Religion	Yes	102 (55.4)	0.67 ± 0.17	−2.53 (0.012) **
		No	82 (44.6)	0.60 ± 0.16	
Intrapersonal characteristics	Smoking	Yes	25 (13.6)	0.59 ± 0.22	−1.12 (0.273)
		No	1599 (86.4)	0.64 ± 0.16	
	Alcohol drinking	Yes	136 (73.9)	0.63 ± 0.16	0.62 (0.536)
		No	48 (26.1)	0.65 ± 0.19	
Interpersonal characteristics	Having a helper	Yes	71 (38.6)	0.58 ± 0.17	−3.82 (<0.001) ***
		No	113 (61.4)	0.67 ± 0.16	
	Living with others	With family	14 (7.6)	0.74 ± 0.08	4.42 (<0.001) ***
		Alone	170 (92.4)	0.62 ± 0.17	

** *p* < 0.01, *** *p* < 0.001.

**Table 2:** Scores between Health Care Service Needs, Sleep Quality, Social Support, and Health-Related Quality of Life of the Participants (*N* = 184).

Factors	Variables	M ± SD	Min.	Max.	Range
Intrapersonal characteristics	Health Care Service Needs	4.98 ± 2.17	0	9	0~9
	Sleep Quality	9.82 ± 3.21	0	19	0~21
Interpersonal characteristics	Social Support	3.24 ± 0.73	1.50	4.75	1~5
Health related Quality of Life		0.64 ± 0.17	0.06	0.95	−1~1

**Table 3:** Effects of Participant’s General, Intrapersonal, Interpersonal Characteristics on Health-Related Quality of Life (*N* = 184).

Factors	Variables	Categories	Model 1				Model 2				Model 3			
			B	β	t	*p*	B	β	t	*p*	B	β	t	*p*
(Constant)			0.59		24.89	<0.001 ***	0.77		15.03	<0.001 ***	0.30		4.31	<0.001 ***
General characteristics	Religion (ref. no)		0.06	0.18	2.55	0.011 *	0.05	0.16	2.25	0.026 *	0.06	0.17	2.97	0.003 **
	Education (ref. no)	Elementary	−0.16	−0.05	−0.59	0.556	−0.03	−0.09	−1.18	0.242	−0.03	−0.09	−1.42	0.159
		Middle	0.12	0.20	2.64	0.009 **	0.07	0.11	1.46	0.147	0.03	0.06	0.88	0.378
		High	0.07	0.12	1.54	0.127	0.02	0.04	0.54	0.592	0.01	0.02	0.25	0.804
Intrapersonal characteristics	Health care service needs						−0.15	−0.21	−2.68	0.008 **	−0.04	−0.05	−0.77	0.445
	Sleep quality						−0.01	−0.15	−1.97	0.050	−0.00	−0.07	−1.21	0.228
Interpersonal characteristics	Having a helper (ref. no)										−0.07	−0.21	−3.60	<0.001 ***
	Living with others (ref. no)										0.06	0.10	1.70	0.090
	Social Support										0.12	0.52	8.78	<0.001 ***
R^2^			0.096				0.170				0.478			
Adjusted R^2^			0.075				0.142				0.451			
F (*p*)			4.73 (0.001) **				7.92 (0.001) **				34.32 (<0.001) ***			

* *p* < 0.05, ** *p* < 0.01, *** *p* < 0.001.

## Data Availability

The data presented in this study are available on request from the corresponding author. The data are not publicly available due to the protection of the privacy of research subjects.
